# The janus-faced biology of GPNMB: from tissue homeostasis to cancer pathogenesis

**DOI:** 10.3389/fmolb.2025.1664764

**Published:** 2025-10-17

**Authors:** Cheng Long, Li Qin Yun, Ma Dai Yuan, Imad Ibrahim Ali Al-Sultan

**Affiliations:** ^1^ Department of Oncology, Affiliated Hospital of North Sichuan Medical College, Nanchong, China; ^2^ School of Graduate Studies, Post Graduate Centre, Management and Science University, Shah Alam, Selangor, Malaysia; ^3^ International Medical School, Management and Science University, Shah Alam, Selangor, Malaysia

**Keywords:** GPNMB, osteoactivin, DC-HIL, tumor microenvironment, immuneescape, prognostic, targeted therapy

## Abstract

Glycoprotein non-metastatic melanoma protein B (GPNMB) is a transmembrane glycoprotein that differentially regulates tissue homeostasis and disease pathogenesis. In physiological contexts, it maintains melanosome biogenesis, osteogenesis, and neuroprotection through domain-specific interactions. Pathologically, tumors exploit GPNMB’s dual mechanisms: membrane-bound isoforms mediate T cell exclusion via DC-HIL/Syndecan-4, while soluble GPNMB(sGPNMB) promote metabolic reprogramming through CD44/NF-κB. Clinically, GPNMB overexpression correlates with poor outcomes, notably demonstrating 40% versus 8% ADC response in high- versus low-expressing TNBC (p < 0.001). Emerging data reveal its crosstalk with HER2/FGFR1 pathways and identify K48-ubiquitination as a therapeutic resistance mechanism. These findings position domain-selective GPNMB targeting as a promising precision oncology strategy.

## 1 Introduction

GPNMB originally identified in melanoma in 1995 (Hoashi et al., 2010), has been recognized as a pleiotropic regulator modulating both physiological homeostasis and disease pathogenesis through its multidomain structure containing an RGD integrin-binding motif, PKD domain, and hemITAM signaling motif (Theos et al., 2013). While essential for melanosome formation and wound healing under physiological conditions, GPNMB is aberrantly overexpressed and functionally co-opted in multiple pathologies (Taya and Hammes, 2018). In oncology, GPNMB overexpression drives malignant progression through three well-characterized mechanisms: (1) immunosuppression via DC-HIL/Syndecan-4-mediated CD8+T-cell exclusion (Xiong et al., 2022). (2) metabolic dysregulation through hepatocyte-secreted sGPNMB in obesity-associated cancers (Gibbons et al., 2022). (3) therapeutic resistance consequent to ADAM10/17-mediated ectodomain shedding (Ott et al., 2014). Notably, domain conservation enables GPNMB to orchestrate parallel pathogenic cascades-activating PI3K/Akt in malignancies while disrupting microglial function in neurodegeneration through shared hemITAM interactions (Diaz-Ortiz et al., 2022). This context-dependent functionality, regulated by cell-type-specific post-translational modifications, establishes GPNMB as a molecular switch governing the balance between tissue maintenance and disease pathogenesis.

## 2 Structural foundations and functional complexity

**FIGURE 1:**
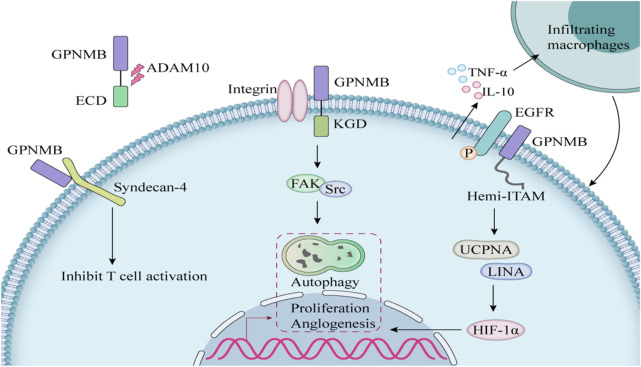
The mechanism of action of GPNMB in cells.

GPNMB is a structurally complex type I transmembrane glycoprotein with diverse physiological and pathological functions mediated through three critical domains (Table 1). The extracellular domain (ECD) harbors an evolutionarily conserved RGD motif (position 452-454) that binds α5β1/αvβ3 integrins (Cancer Genome Atlas Research Network, 2014), facilitating metastatic dissemination in breast and prostate cancer models (Fiorentini et al., 2014). The intracellular domain (ICD) contains a hemITAM motif that recruits SYK kinases, activating pro-survival PI3K/Akt/mTOR and MEK/ERK pathways in glioblastoma (Bao et al., 2016). ADAM10/17-mediated ectodomain shedding generates sGPNMB (Xie et al., 2019), and this sGPNMB promotes angiogenesis by inducing VEGFR2 phosphorylation (Rose et al., 2010) (Figure 1).

**TABLE 1:** GPNMB domain functions and disease associations.

Author(s)-Year	Disease association	Domain	Function
Maric et al. (2013)	Breast cancer metastasis	Extracellular	RGD motif binds α5β1/αvβ3 integrins, activating MMP-2/9
Kloosterman et al. (2024)	Glioblastoma immunosuppression	Extracellular	Interacts with CD44, promoting cholesterol transfer to tumor cells
Elhinnawi et al. (2024)	TNBC progression	Intracellular	Binds FGFR1, activating PI3K-AKT pathway
Oyewumi et al. (2016)	NSCLC angiogenesis	sGPNMB	ADAM10-mediated shedding, stimulating VEGF signaling

These functions are precisely regulated by post-translational modifications: S546 phosphorylation enhances amyloid-β phagocytosis (Taya and Hammes, 2018), K48-linked ubiquitination reduces protein half-life (Diaz-Ortiz et al., 2022), and hypoglycosylation at N275/N350 promotes melanoma cell surface retention (Hoashi et al., 2010; Theos et al., 2013).

## 3 Dual physiological and pathological roles

In physiological contexts, GPNMB maintains tissue homeostasis through IL-4Rα/STAT6-mediated M2 polarization while suppressing NF-κB activation (Saade et al., 2021; Lazaratos et al., 2022). It accelerates wound healing by 41% through FGF2 upregulation (Silva et al., 2018) and enhances osteoblast differentiation (Maric et al., 2013). As a hepatokine, promotes WAT lipogenesis via liver-adipose tissue crosstalk, exacerbating obesity and insulin resistance, while its inhibition improves metabolic disorders (Chung et al., 2011).

In cancer pathogenesis, GPNMB mediates immune evasion via MDSC recruitment and MHC-I downregulation (Lazaratos et al., 2022). It drives metastasis through MMP-2/9 activation and ZEB1-mediated EMT (Fiorentini et al., 2014; Maric et al., 2015). GPNMB confers chemoresistance through Bcl-xL upregulation and diminishes PD-1 inhibitor efficacy (Ott et al., 2014), establishing its dual roles in tissue homeostasis and disease pathogenesis through conserved structural and regulaty mechanisms.

## 4 The role mechanism of GPNMB in non-tumor conditions

**FIGURE 2:**
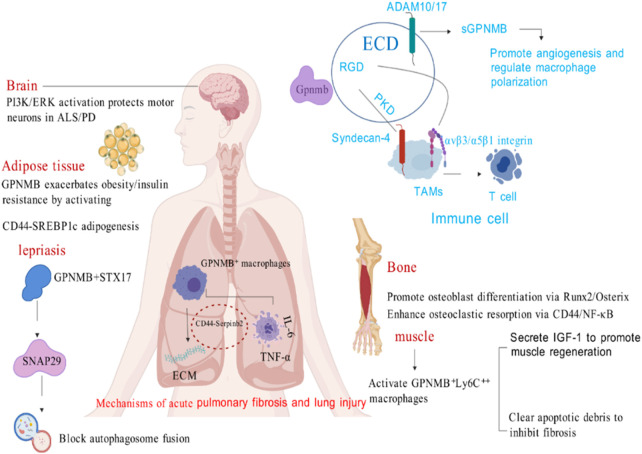
The role of GPNMB in non-tumor conditions (Created with BioGDP.com).

GPNMB is expressed in various normal tissue cells (including the embryonic nervous system, embryonic nephron units, basal layer of the skin, hair follicle stem cells, osteoblasts, osteoclasts, macrophages, and retinal pigment epithelial cells) as well as in other tissues (Ponzetti and Rucci, 2021), and it plays distinct roles in different structural contexts (Figure 2).

### 4.1 Immune system

GPNMB serves as a critical immunomodulator through its dual receptor-ligand interactions and cell-type specific expression. In innate immunity, it is constitutively expressed in dendritic cells (DCs), macrophages, and eosinophils where it (1) recognizes fungal antigens to enhance antimicrobial defense (Chung et al., 2007a), (2) mediates DC-endothelial adhesion via RGD domain binding, (3) suppresses T-cell activation through PKD domain interaction with Syndecan-4 on T cells (Chung et al., 2007b). GPNMB orchestrates anti-inflammatory responses by simultaneously inhibiting pro-inflammatory cytokines while potentiating immunosuppressive factors (Zhou and Du, 2021). This regulatory balance is exemplified in macrophage polarization, where GPNMB deficiency exacerbates IFN-γ/LPS-induced inflammation, and in tissue repair where it recruits M2 macrophages and mesenchymal stem cells to promote wound healing (Gong et al., 2019; Chung et al., 2011). The protein’s developmental significance is evidenced in eosinophil maturation, showing time-dependent expression peaking at day 12 of CD34^+^ progenitor differentiation coincident with MBP1 expression - a process abrogated by anti-GPNMB antibodies or RNAi (Hwang et al., 2017).

GPNMB’s immunoregulatory functions are hijacked in disease states, particularly cancer and autoimmunity. In melanoma, tumor-derived GPNMB engages Syndecan-4 on T cells to blunt antitumor immunity, with knockout studies demonstrating >50% reduction in B16F10 tumor growth (Chung et al., 2011). Clinically, suppressed GPNMB expression correlates with susceptibility to chronic inflammatory conditions including periodontitis, nephritis, and vitiligo (Zhou and Du, 2021). These pathological effects stem from GPNMB’s bifunctional capacity to: (1) induce tolerogenic DC phenotypes via TGF-β signaling (Schwarzbich et al., 2012), and (2) modulate monocyte activation thresholds as shown by enhanced inflammatory responses in GPNMB-mutant mice (Zhang et al., 2012). Therapeutically, GPNMB’s time-restricted expression during immune cell development (Hwang et al., 2017) and its membrane-to-soluble transition mediated by ADAM proteases (Chung et al., 2011) offer unique targeting windows. Current evidence positions GPNMB at the intersection of immunometabolism and stromal remodeling, with its RGD and PKD domains serving as actionable targets to disrupt tumor-immune crosstalk (Chung et al., 2007a; Chung et al., 2007b).

### 4.2 Adipose tissue

GPNMB, a type I membrane glycoprotein, is structurally characterized by ECD, a transmembrane domain, and a cytoplasmic domain. It is most highly expressed in adipose and skin tissues, with notable expression also in the liver and long bones. A key functional feature is the hydrolyzable ECD, which can be released into the bloodstream to exert systemic effects—this property underpins its role as a signaling mediator across multiple physiological and pathological contexts.

Functionally, GPNMB shapes macrophage behavior in obesity: while not induced by typical polarization cues, it enhances IL-4-mediated arginase-1 induction, promoting macrophage polarization toward an anti-inflammatory phenotype and potentially aiding tissue repair. This is critical for mitigating obesity-related metabolic disorders, as dysregulated inflammation in white adipose tissue is a hallmark of such conditions. Beyond adipose tissue, GPNMB acts as a key mediator of liver-adipose crosstalk.

In obese models, WAT expansion triggers a lysosomal biosynthesis program in ATMs rather than classical inflammation. When adipocyte lipid storage capacity is exceeded, lysosomal lipid accumulation occurs, inducing GPNMB. Here, GPNMB operates through two mechanisms: membrane-bound GPNMB remains on anti-inflammatory macrophages, while sGPNMB from pro-inflammatory macrophages binds CD44 to inhibit NF-κB signaling, reducing macrophage inflammatory capacity and alleviating WAT inflammation. Additionally, sGPNMB activates sterol regulatory element-binding protein 1c in adipocytes via CD44, promoting lipogenesis-explaining its positive correlation with BMI and highlighting its dual, context-dependent role in obesity.

### 4.3 Skin

GPNMB plays an important role in the skin, where it is expressed in normal human epidermal keratinocytes and is involved in cell adhesion and the survival of melanocytes. The cytokines IFN-γ and IL-17A can inhibit the expression of GPNMB, further indicating its potential role in skin depigmentation disorders (Biswas et al., 2020). GPNMB as a marker of melanocytes, is involved in all stages of melanosome maturation. Silencing the GPNMB gene weakens the formation of early melanosomes mediated by UVB, further proving its importance in melanosome formation (Le Borgne et al., 2001). Using siRNA interference technology, the study found that silencing the expression of GPNMB significantly reduced the number of melanosomes in PIG1 melanocytes, indicating that GPNMB is essential for melanosome formation. Experimental results showed that UVB radiation could upregulate the mRNA and protein expression of GPNMB, and that the expression of GPNMB gradually increased after UVB exposure. Furthermore, the relationship between the function of GPNMB and the regulation by MITF remains unclear; however, the structural characteristics of GPNMB are similar to those of the melanosome-specific structural protein Pmel17, suggesting that it may play an important role in the maturation of melanosomes (Zhang et al., 2012). Abnormal expression of GPNMB is not only associated with skin diseases such as dyschromatosis, amyloidosis, and vitiligo, but it can also lead to melanoma. Therefore, further research on GPNMB and targeted therapies could provide more effective treatment strategies for skin-related diseases.

GPNMB plays a pivotal role in regulating autophagy and intracellular bacterial infections, as evidenced by its significantly elevated expression in macrophages from lepromatous leprosy (L-Lep) patients. During infection, GPNMB mechanistically impairs xenophagy by binding to autophagosomal STX17, reducing its N296 site glycosylation, and promoting SNAP29 degradation, thereby preventing formation of the STX17-SNAP29-VAMP8 SNARE complex required for autophagosome-lysosome fusion. This disruption of autophagic flux extends beyond *Mycobacterium leprae*, as GPNMB deficiency broadly inhibits proliferation of diverse intracellular bacteria in human macrophages, demonstrating its universal role in regulating intracellular bacterial clearance through autophagy modulation (Yan et al., 2025).

### 4.4 Brain

GPNMB exerts neuroprotective effects on damaged brain tissue. It is expressed in normal brain tissue, with increased expression observed following central nervous system injury. In astrocytes, GPNMB induces survival signals in nearby motor neurons through the PI3K and MEK/ERK signaling pathways, improving neurodegeneration induced by atrophy. Conversely, the degradation of phosphorylated GPNMB mediated by polyubiquitination ultimately leads to motor neuron death (Tanaka et al., 2012). Furthermore, the loss of GPNMB leads to impaired internalization of aSyn fibrils, thereby affecting neuronal function (Diaz-Ortiz et al., 2022).

These mechanisms jointly affect the process of neuroinflammation (Gillett et al., 2023). The gene transcripts such as KLHL7, KLHL7 - AS1, NUPL2, and AC005082.12, affecting their expression in different tissues. Although the function of GPNMB is unknown, it is related to diseases such as ALS and participates in various molecular functions. Other genes at the same locus also have their respective cellular functions. The regulation at this locus is tissue and cell specific. GPNMB is highly expressed in glial cells and lowly expressed in neurons. Although the increased expression of GPNMB in the brain region is associated with the risk of PD, the role of other genes at this locus in the pathogenesis of PD cannot be excluded (Murthy et al., 2017).

### 4.5 Lung

In the research related to lung diseases, GPNMB mainly serves as a marker of the recruited macrophage subset. This subset is commonly present in the lung injury models induced by LPS and bleomycin and represents a conserved response to tissue injury. Gpnmb RecAM is enriched with fibrosis-related genes such as Spp1. It has a relatively high expression level even at the steady state, and its expression trends are similar during the inflammatory periods in different injury models, suggesting that this might be a general response of macrophages to injury or inflammation rather than being specific to fibrosis. In acute lung injury (ALI), GPNMB plays an important role by regulating the mitochondrial-mediated apoptotic pathway. Hyperoxia-induced acute lung injury (HALI) can lead to structural and functional damage of the lung tissue. GPNMB has been identified as a key gene in HLI, and its expression is abnormally high in HLI tissues. *In vitro* experiments, MLE-12 cells were treated to construct an ALI model. It was found that GPNMB knockdown could increase cell viability and reduce apoptosis. This is because the treatment alters the expression of apoptosis-related proteins, and GPNMB knockdown can reverse this change and regulate the mitochondrial-mediated apoptotic pathway. Meanwhile, GPNMB knockdown inhibits the production of intracellular ROS induced by, prevents the depolarization of mitochondrial membrane potential, improves mitochondrial function, and reduces mitochondrial-dependent apoptosis. erefore, GPNMB plays a crucial role in acute lung injury by affecting apoptosis-related proteins, ROS, and mitochondrial membrane potential (Wang et al., 2024). In pulmonary fibrosis, macrophage-derived GPNMB shows significant enrichment in fibrotic ECM with a 4.2-fold increase, where it activates fibroblasts through the CD44/Serpinb2 signaling axis. This leads to marked promotion of collagen deposition, as evidenced by 2.1-fold upregulation of Col1A1. Notably, this fibrogenic mechanism operates in parallel with GPNMB’s established immunosuppressive activity mediated through Syndecan-4, collectively constituting dual pro-fibrotic pathways that drive disease progression (Wang et al., 2023). In other lung-related diseases, GPNMB also plays an important role through complex mechanisms. Intranasal administration of β-glucan can induce the production of ApoE^+^CD11b^+^ MoAMs, in which GPNMB is highly expressed. The cell source is Ly6c^+^ monocytes in the bone marrow and is dependent on CCR2. Functionally, ApoE^+^CD11b^+^ AMs release a large amount of IL-6 upon LPS restimulation, and at the same time, metabolic reprogramming increases glycolysis and phagocytic activity, which is related to GPNMB. In the regulatory mechanism, β-glucan regulates the production of GPNMB-related cells through the Dectin-1/CARD9 signaling axis, and ApoE regulates their differentiation and survival through paracrine manner. The deficiency of ApoE affects cholesterol metabolism and M-CSF secretion, thereby affecting GPNMB-related cells. The mechanism of GPNMB in lung diseases is a complex network involving multiple aspects and is of great significance for disease research (Theobald et al., 2024).

### 4.6 Skeleton and muscle

GPNMB exhibits remarkable tissue specificity and functional extensibility in its biological functions. Its core regulatory role in benign bone homeostasis allows it to directly regulate the differentiation and function of osteoblasts (Rose et al., 2017). By activating osteogenesis-related signaling pathways, GPNMB positively promotes bone formation, thereby providing a molecular basis for bone development and repair (Abdelmagid et al., 2008). Furthermore, transgenic mouse experiments have revealed its property of bidirectionally regulating bone metabolism (Sheng et al., 2012), which enables a strong association with its targeted application in the field of cancer therapy through molecular functional consistency (Tse et al., 2006).

In bone tissue, GPNMB can promote the differentiation of osteoclasts and is an important osteogenic factor (Tomihari et al., 2010). Its absence negatively affects osteoclast differentiation and the mineralization of bone matrix. OA also known as GPNMB, is a glycoprotein that is highly expressed during osteoblast differentiation and has two isoforms: one is transmembrane, and the other is secreted into the conditioned medium. Studies have shown that the mature 115 kDa OA isoform is primarily found in the membranous fraction and is also present in the cytoplasm of osteoblasts. OA reaches its maximum expression during the third week of culture and is highly glycosylated. Retinoic acid stimulates the mannosylation of OA, while tunicamycin inhibits the incorporation of N-glycans into OA. Treatment with anti-OA antibodies results in decreased osteoblast differentiation, whereas OA overexpression promotes osteoblast differentiation and function. Additionally, studies using OA mutant mice indicate that OA is crucial for terminal osteoblast differentiation and mineralization, as the differentiation capacity of mutant osteoblasts is significantly lower than that of wild-type osteoblasts. Overall, OA is considered a positive regulator of osteogenesis (Manganelli et al., 2023). Another study indicates that the overexpression of OA promotes the maturation of osteoclasts and their bone resorption activity, leading to increased bone loss. By using a transgenic mouse model, it was found that OA affects bone metabolism by enhancing the function of osteoclasts rather than their quantity (Turre et al., 2014).

To sum up, the above summarizes the role of GPNMB under normal physiological conditions, which is summarized in Table 2, while the core mechanisms are conserved across tissues, their functional outcomes diverge as summarized in Table 3. This functional divergence manifests as three distinct pathological patterns: (1) metabolic reprogramming in adipose tissue through IL-4/arginase-1 axis, (2) immunosuppressive niche formation in tumors via DC-HIL/Syndecan-4 interaction, and (3) fibrotic ECM remodeling in lung through CD44/Serpinb2 activation.

**TABLE 2:** Role of GPNMB in normal physiological conditions across different tissues.

Tissue/System	Core role
Immune system	Regulates inflammation, maintains immune homeostasis, and inhibits T cell activation (Silva et al., 2018; Chung et al., 2007b; Biswas et al., 2020; Le Borgne et al., 2001)
Adipose tissue	Alleviates white adipose tissue inflammation and regulates lipogenesis (Ponzetti and Rucci, 2021; Chung et al., 2007a)
Skin	Promotes melanosome maturation and regulates autophagy (Chung et al., 2007b; Zhou and Du, 2021; Gong et al., 2019; Chung et al., 2011)
Brain	Provides neuroprotection and enhances microglial phagocytosis (Diaz-Ortiz et al., 2022; Hwang et al., 2017; Schwarzbich et al., 2012; Le Borgne et al., 2001)
Lung	Mitigates acute lung injury and inhibits pulmonary fibrosis (Zhang et al., 2012; Yan et al., 2025; Tanaka et al., 2012)
Bone & Muscle	Enhances osteoblast differentiation and promotes muscle regeneration (Tomihari et al., 2010; Turre et al., 2014; Manganelli et al., 2023)

**TABLE 3:** Conserved mechanisms vs. tissue-specific outcomes of GPNMB.

Core mechanism	Physiological context	Pathological exploitation	Key modulators	Therapeutic implications
CD44/NF-κB signaling	Adipose: M2 macrophage polarization	Obesity: Lipogenesis	sGPNMB	Anti-obesity drugs (Lazaratos et al., 2022)
DC-HIL/Syndecan-4	Skin: T cell tolerance	Melanoma: T cell exclusion	ADAM10	CR011-vcMMAE ADC (Tomihari et al., 2010)
Integrin RGD motif	Bone: Osteoblast differentiation	Metastasis: MMP-2/9 activation	α5β1/αvβ3 integrins	Cilengitide analogs (Fiorentini et al., 2014)
HemITAM/SYK kinase	Brain: Microglial phagocytosis	GBM: PI3K/Akt hyperactivation	N-linked glycosylation	SYK inhibitors (ENTA-727) (Elhinnawi et al., 2024)

## 5 Study of GPNMB in different tumor types

**FIGURE 3:**
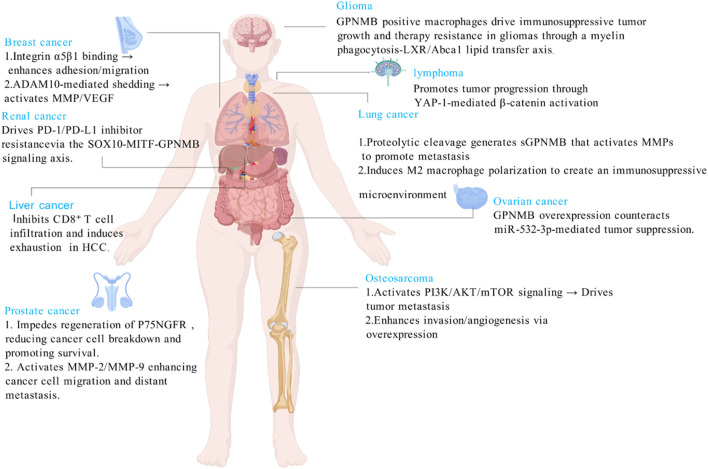
GPNMB drives cancer via tissue-specific metastatic and immunosuppressive programs.

GPNMB has transformed from an initially identified tumor suppressor to a key oncogenic driver. Unlike its role in maintaining homeostasis in non-tumor diseases, GPNMB mediates immune suppression by promoting the polarization of tumor-associated macrophages toward the M2 phenotype and metastatic colonization by enhancing tumor cell metabolism and invasive capacity through aberrant activation of common pathways such as CD44/NF-κB and mTORC1 in tumors (Lazaratos et al., 2022). It promotes malignant progression in various cancers including breast cancer and head and neck squamous cell carcinoma by enhancing proliferation, invasion, metastasis, and inducing EMT and stemness. Its overexpression correlates with poor prognosis, making it a promising pan-cancer therapeutic target (Figure 3).

### 5.1 Melanoma

GPNMB is highly expressed in melanoma, particularly showing co-expression with melanocyte-related genes, and serves as a critical target for the antibody-drug conjugate CR011-vcMMAE. *In vivo* studies demonstrate that CR011-vcMMAE exhibits dose-dependent tumor growth inhibition, with low doses suppressing tumor progression and high doses inducing complete regression (Williams et al., 2010). Genetic ablation of GPNMB significantly reduces pulmonary metastatic lesions in B16F10 melanoma models and decreases melanin deposition at metastatic sites (Li et al., 2019). Mechanistically, GPNMB mediates immune evasion through the DC-HIL/syndecan-4 axis, inhibiting T-cell activation and IFN-γ production while promoting an immunosuppressive microenvironment via recruitment of myeloid-derived suppressor cells (Kawasaki et al., 2022; van Schaik et al., 2024). Notably, early-stage uveal melanoma shows an 85.7% positivity rate for GPNMB expression, highlighting its therapeutic potential (Wang et al., 2021a). These findings establish GPNMB’s dual oncogenic roles in melanoma progression: as a pro-metastatic factor regulating melanin synthesis and metastatic niche formation, and as an immune checkpoint mediator via the DC-HIL pathway, making it an ideal target for both antibody-drug conjugates and combination immunotherapy. However, its therapeutic efficacy in metastatic uveal melanoma requires further validation.

### 5.2 Head and neck cancer

GPNMB-positive cells exhibit cancer stem cell characteristics in head and neck squamous cell carcinoma (HNSCC), the GPNMB positivity rate is expected to be approximately 15%. Promoting EMT and being associated with poorer prognosis (van Eijk and Aerts, 2021). Patients with high GPNMB expression have lower survival rates and progression-free survival, and show greater resistance to chemotherapy and radiotherapy. To compare the expression levels of GPNMB with those of CSC makers and EMT markers on the same specimens, this study immunostained serial sections of biopsy specimens with the anti-GPNMB antibody along with the antibodies against CSC markers including SOX2 and Nanog and the antibody against EMT markers including Snail/Slug. As shown in Table 3, while the HNSCC expressing a high level of GPNMB gave intensive signals of both CSC markers and EMT markers, that expressing no or a low level of GPNMB did not. This result thus indicates that GPNMB behaves as a marker of both CSC and EMT (Erbani et al., 2022). Another study aimed to evaluate the potential of GPNMB and VEGF as fluorescent imaging targets in primary head and neck squamous cell carcinoma and lymph node metastases, and compare them with epidermal growth factor receptor. A total of 38 samples of untreated HNSCC primary tumors and corresponding synchronous lymph node metastases were selected. Protein expression was assessed by immunohistochemical staining. The results showed that the expression of GPNMB in both primary tumors and lymph node metastases was 100%, and the percentage of tumor cells expressing GPNMB was higher than that of EGFR. The positive expression rates of VEGF and EGFR in primary tumors were 92% and 87%, respectively, and those in lymph node metastases were 87% and 84%, respectively. The expression of GPNMB and EGFR in primary tumors and lymph node metastases was positively correlated, while VEGF showed no correlation. The conclusion of the study is that the expression of GPNMB in untreated HNSCC primary tumors and lymph node metastases is higher than that of EGFR, and the correlation of its expression between the two makes GPNMB a promising target for fluorescent imaging in HNSCC (Nichol et al., 2016).

Oral squamous cell carcinoma (OSCC) is a common malignant tumor in the head and neck, accounting for 90% of oral malignancies. The mechanism of the occurrence and development of OSCC is complex. Studies have found that the tumor microenvironment is one of the main factors for tumor invasion. *In vitro* studies have shown that macrophages in the OSCC microenvironment can release sGPNMB. Research has confirmed that GPNMB plays an important role in the expansion of cancer stem cells, prolonging cell survival, and promoting the metastatic phenotype *in vivo*. CD44 is one of the functional receptors of GPNMB and is considered as a surface marker of cancer stem cells, interacting with tumor-associated macrophages (Berghoff et al., 2022).

Recent studies on malignant brain tumors have found that GPNMB-high macrophage clusters, such as MG3-GPNMB and MDM3-GPNMB belong to lipid-laden macrophages (Garofano et al., 2021; Kloosterman et al., 2024). These cells form through the phagocytosis of myelin debris, a process accompanied by cholesterol accumulation and epigenetic remodeling like the elevation of H3K27me3 levels (Maric et al., 2019). They display immunosuppressive characteristics, including the upregulation of PD-L1 and CD39 expression, and co-localize with mesenchymal-like tumor cells in hypoxic niches such as pseudopalisading necrosis areas, with their proportion significantly increasing in recurrent tumors. GPNMB-positive macrophages promote tumor cell proliferation and sphere-forming capability by transferring myelin-derived cholesterol and fatty acids to tumor cells through an LXR/Abca1-dependent pathway (Kanematsu et al., 2015). The high expression of their characteristic genes correlates with poor prognosis in glioblastoma patients and is associated with MES subtypes and resistance to immune checkpoint therapy in human tumors (Rose et al., 2007). As key molecular markers of the metabolic crosstalk among myelin, macrophages, and tumor cells, their expression is regulated by the epigenetic remodeling induced by myelin phagocytosis, shows a negative correlation with lymphocyte infiltration, and possibly maintains an immunosuppressive microenvironment by inhibiting MHC II expression (Kloosterman et al., 2024).

### 5.3 Breast cancer

GPNMB emerges as a key oncogenic driver in breast cancer, particularly in aggressive subtypes, through multifaceted mechanisms. In triple-negative breast cancer (TNBC), GPNMB overexpression correlates with poor prognosis and demonstrates therapeutic vulnerability, as evidenced by CDX-011 (anti-GPNMB ADC) achieving 40% objective response rates. Mechanistically, GPNMB promotes tumor progression through: 1) RGD domain-mediated binding to α5β1 integrin, enhancing fibronectin adhesion and metastatic potential (Maric et al., 2015); 2) ADAM10-dependent ectodomain shedding, generating sGPNMB that stimulates angiogenesis via VEGF/NRP-1 signaling and endothelial cell migration; 3) direct interaction with FGFR1 in TNBC, activating PI3K-AKT pathway and enhancing sphere-forming capacity (Elhinnawi et al., 2024).

Notably, GPNMB exhibits subtype-specific functions. In basal-like breast cancer, while non-oncogenic alone, it synergizes with Wnt-1 to accelerate tumorigenesis via PI3K/AKT/β-catenin axis activation, increasing proliferation while reducing apoptosis (Maric et al., 2019). In HER2 positive GPNMB depletion enhances trastuzumab sensitivity by upregulating HER2/EGFR phosphorylation through ERK/MAPK pathway modulation (Kanematsu et al., 2015).

The protein demonstrates unique spatial regulation in metastasis. In bone metastasis, GPNMB promotes osteotropism by enhancing migratory/invasive capacities while suppressing T-cell activation (Rose et al., 2007). At metastatic niches, GPNMB + regulatory macrophages expressing TREM2 accumulate at invasive fronts, creating immunosuppressive microenvironments that facilitate immune evasion (Yofe et al., 2023).

Crucially, GPNMB marks cancer stem cells in breast tumors, inducing EMT transcription factors and sphere-forming capacity without proliferation marker expression, suggesting a dormancy-associated stemness program (Okita et al., 2018). These findings position GPNMB as both a prognostic biomarker and therapeutic target, particularly for TNBC and basal-like subtypes, with current clinical evaluation focusing on ADCs and combinatorial strategies targeting its multifaceted roles in metastasis, angiogenesis, and immune modulation.

### 5.4 Lung cancer

Mounting evidence establishes GPNMB as a critical player in lung cancer progression through dual oncogenic mechanisms. Clinically, elevated GPNMB expression serves as a potent negative prognostic indicator across subtypes-SCLC patients with high serum levels show significantly reduced median OS (Cancer Genome Atlas Research Network, 2014), while metastatic lymph nodes exhibit 3.2-fold higher expression than primary tumors (Liu et al., 2024). Mechanistically, ADAM10-mediated shedding generates sGPNMB that drives tumor cell migration and invasion through MMP activation (Oyewumi et al., 2016), while simultaneously shaping an immunosuppressive TME marked by M2 macrophage infiltration and CD44^+^ immune cell accumulation (Boulle et al., 2020). Notably, GPNMB shows consistent overexpression in rare lymphangioleiomyomatosis lesions (Tuo et al., 2024), expanding its diagnostic utility.

### 5.5 Prostatic cancer

Prostate cancer is the most common cancer among men in Europe and the United States, and its mortality rate ranks second among all male cancers. In the early stage, it can be treated by surgery and anti-androgen therapy (Mouraviev et al., 2009). However, this regimen is basically ineffective in castration-resistant prostate cancer. Therefore, more targeted therapies for prostate cancer are needed. During the screening of genes related to the metastasis and invasion of prostate cancer, abnormal expression of GPNMB was found. In prostate cancer cells DU145 and PC3 with high expression of GPNMB, exogenous addition of nerve growth factor (NGF) can induce the regeneration of the NGF receptor P75NGFR, and P75NGFR is related to the degradation of cancer cells. Meanwhile, the expression level of GPNMB was found to decrease. This indicates that in prostate cancer, GPNMB may reduce the degradation of cancer cells by affecting P75NGFR, thereby promoting their proliferation.

GPNMB binds to integrins such as α5β1/αvβ3 via the RGD motif in its extracellular domain, activating downstream MMP family members including MMP-2/9 to enhance tumor cells’ ability to degrade the extracellular matrix. This common mechanism functions in prostate cancer invasion, breast cancer bone metastasis, and lung cancer metastasis, serving as the core pathway through which GPNMB promotes cross-cancer metastasis (Fiorentini et al., 2014; Kloosterman et al., 2024).

### 5.6 Ovarian cancer

Epithelial ovarian cancer is the most common type of ovarian cancer with a relatively high mortality rate. Through immunohistochemical analysis of tissue samples and real-time quantitative PCR for biopsy of living tissues, it has been found that compared with normal tissues, GPNMB is highly expressed in various types of epithelial ovarian cancer, such as serous carcinoma and endometrioid carcinoma. When studying the relationship between the expression of GPNMB and the clinical characteristics of epithelial ovarian cancer, it was discovered that its expression level is closely related to the staging of ovarian cancer. Moreover, both the residual tumor after treatment and lymph node metastasis are associated with GPNMB. Among them, the staging of ovarian cancer, the residual tumor, and the expression of GPNMB can all serve as independent prognostic indicators for ovarian cancer. This suggests that GPNMB may be a target for the diagnosis and treatment of ovarian cancer (Qiong Ma et al., 2018).

It was observed that the forced expression of miR-532–3p exerted inhibitory effects on the proliferation, migration, and invasion of ovarian cancer cells *in vitro*. Moreover, it also hindered the growth of tumors in nude mice. Through RNA sequencing, it was identified that 299 mRNAs exhibited downregulated expression in ovarian cancer cells with overexpressed miR-532-3p. Subsequently, bioinformatic analysis suggested that GPNMB, which belongs to type I membrane glycoprotein, was likely to be the target of miR-532–3p. In the ovarian cancer cells where miR-532-3p was overexpressed, the levels of GPNMB were decreased both at the RNA and protein levels. The dual-luciferase reporter assay further confirmed that GPNMB was indeed the target of miR-532-3p.When GPNMB was interfered with, it led to the inhibition of several key aspects in ovarian cancer cells, including their proliferation, migration, invasion, glucose consumption, and lactate production. Additionally, knocking down GPNMB resulted in a reduction in the protein level of HIF-1α, while having no impact on the mRNA level of HIF-1α.Most notably, the overexpression of GPNMB was able to reverse the antitumor effect that miR-532–3p had exerted, indicating a significant relationship between them in regulating the biological behaviors and functions of ovarian cancer cells (Tuo et al., 2022).

### 5.7 Osteosarcoma

Osteosarcoma, a relatively common bone tumor among both children and adults, has an extremely high degree of malignancy and strong abilities of invasion and metastasis, posing a serious threat to the life and health of patients. Moreover, the disability caused by surgery has brought heavy economic and psychological burdens to families and society. Although the treatment methods for osteosarcoma in the current medical field have been continuously developed and optimized, the survival rate of patients remains at a relatively low level (Vasquez et al., 2016).

Numerous studies have shown that during the occurrence and development of osteosarcoma, the PI3K/Akt/mTOR signaling pathway is often in an activated state. When this signaling pathway is inhibited, the proliferation and invasion abilities of osteosarcoma cells can be significantly weakened. Further research has found that the PI3K/Akt/mTOR signaling pathway also has the function of regulating numerous downstream tyrosine kinase receptors, among which the representative one is the insulin-like IGF-1 receptor (Yuan and Cantley, 2008). Whether this receptor is activated or not will have a profound impact on the proliferation and migration processes of cells. Specifically, the IGF-1 receptor participates in regulating key aspects such as the cell cycle and cytoskeleton remodeling through a series of complex intracellular signal transduction mechanisms, thereby influencing the proliferation and migration characteristics of cells (Ludwig et al., 2011).

When a comparative analysis was conducted between osteosarcoma tissues and normal tissues, it was found that the mRNA level and protein level of GPNMB in osteosarcoma tissues were both significantly upregulated compared with those in normal tissues. To deeply explore the association between GPNMB and the biological behaviors of osteosarcoma cells, researchers carried out gene knockout experiments in osteosarcoma cells MG63 and U2OS. The results showed that after the GPNMB gene was knocked out, the proliferation and migration abilities of the cells exhibited a significant downward trend. Meanwhile, when detecting the expression levels of proteins related to the PI3K/Akt/mTOR signaling pathway, it was found that the levels of key proteins such as p-PI3K, PI3K, p-Akt, Akt, p-mTOR, and mTOR were all significantly downregulated, which meant that the PI3K/Akt/mTOR signaling pathway was also significantly inhibited. On this basis, researchers further carried out *in vitro* experiments by exogenously adding the activator IGF-1 of the PI3K/Akt to the cultured osteosarcoma cells. Subsequent detection results indicated that the expression levels of the above-mentioned key proteins showed an upward trend, and the degree of inhibition on the PI3K/Akt/mTOR signaling pathway was accordingly reduced. As a result, this pathway was activated again, and correspondingly, the impact on the proliferation and metastasis abilities of osteosarcoma cells returned to the previous level (Jin et al., 2018).

Based on the comprehensive analysis of the above experimental results, it can be concluded that GPNMB can play a regulatory role in the proliferation and migration processes of osteosarcoma cells by acting on the PI3K/Akt/mTOR signaling pathway. In view of this important finding, the feasibility of developing new therapeutic drugs or treatment regimens targeting GPNMB has begun to be explored, bringing new hope for the future treatment of osteosarcoma.

Progress Compared to normal tissues, GPNMB is expressed at higher levels in cancer tissues. Its normal physiological functions include anti-inflammatory effects, neuroprotection, and involvement in melanosome synthesis. However, in tumor tissues, GPNMB can promote the growth, proliferation, and metastatic invasion of various cancer cells, as well as enhance angiogenesis in the surrounding tumor environment. Consequently, GPNMB has emerged as a potential therapeutic target for multiple cancers, including melanoma, breast cancer, and osteosarcoma.

### 5.8 Others cancers

Studies have confirmed that serum GPNMB levels significantly increase in renal cell carcinoma patients with acquired resistance to immune checkpoint inhibitors. The mechanism involves PDL1 receptor activating SOX10 via intracellular signaling, which in turn drives the dysregulation of microphthalmia-associated transcription factor (MITF) pathway to promote GPNMB overexpression, forming the SOX10-MITF-GPNMB signaling cascade responsible for acquired resistance. Plasma cell-free RNA analysis in clinical samples shows activation of the SOX10-MITF-GPNMB axis in acquired resistance patients, consistent with elevated GPNMB levels. Targeting GPNMB reverses immune checkpoint inhibitors resistance in mouse models, suggesting that GPNMB can serve as both a prognostic marker for a potential therapeutic target in RCC patients (Chung et al., 2025). In diffuse large B-cell lymphoma, silencing GPNMB can inhibit the nuclear translocation of β-catenin protein and weaken the malignant phenotype of tumor cells, which promotes tumor progression through the Wnt pathway (Wang et al., 2021b). However, for other hematologic malignancies such as leukemia, multiple myeloma, and other types of lymphoma, research on GPNMB is still in its infancy. There is a lack of comprehensive studies on how GPNMB is expressed, its functional role, and its potential as a therapeutic target.

Another liver cancer study show that GPNMB expressed by tumor endothelial cells plays a significant role. GPNMB is highly expressed in TECs and is associated with tumor growth and the exhaustion of tumor-infiltrating CD8^+^ T cells. GPNMB affects the migration and infiltration of CD8^+^ T cells from tumor vessels into tumor tissues, leading to the functional exhaustion of CD8^+^ T cells, manifested as increased expression of inhibitory receptors, decreased IFN-γ production, and ROS accumulation. Downregulating GPNMB by siRNA can inhibit the proliferation and migration of TECs. *In vivo*, it slows down tumor growth, increases the number of tumor-infiltrating CD8^+^ T cells, reduces the proportion of exhausted cells, increases IFN-γ production, and decreases ROS accumulation. Therefore, GPNMB is crucial in the immune escape of liver cancer and is a potential therapeutic target. It is expected to enhance the treatment effect of liver cancer in combination with immunotherapies such as anti-PD-1 treatment (Zhang et al., 2019).

Collectively, GPNMB exerts context-dependent roles across various tumors—while it drives tumor progression via mechanisms like immunosuppressive niche formation (Table 4), metastatic promotion, and metabolic reprogramming in most cancer types.

**TABLE 4:** Role of GPNMB in various cancer types.

Cancer type	Core oncogenic role	Prognostic/Therapeutic relevance
Melanoma	Mediates immune evasion and promotes metastasis (Williams et al., 2010; Li et al., 2019; Kawasaki et al., 2022; van Schaik et al., 2024; Wang et al., 2021a)	Correlates with poor prognosis; ADC CR011-vcMMAE induces tumor regression (Williams et al., 2010; Li et al., 2019; Kawasaki et al., 2022; van Schaik et al., 2024; Wang et al., 2021a)
HNSCC	Drives cancer stemness/EMT and enhances chemo-radioresistance (van Eijk and Aerts, 2021; Erbani et al., 2022; Nichol et al., 2016)	Associated with reduced survival; serves as a potential imaging target (van Eijk and Aerts, 2021; Erbani et al., 2022; Nichol et al., 2016)
TNBC	Enhances metastasis and promotes angiogenesis (Elhinnawi et al., 2024; Berghoff et al., 2022; Maric et al., 2019; Kanematsu et al., 2015; Rose et al., 2007)	Linked to poor prognosis; ADC CDX-011 achieves 40% ORR (Elhinnawi et al., 2024; Berghoff et al., 2022; Maric et al., 2019; Kanematsu et al., 2015; Rose et al., 2007)
Lung Cancer	Boosts cell invasion and forms immunosuppressive microenvironment (Cancer Genome Atlas Research Network, 2014; Yofe et al., 2023; Okita et al., 2018; Liu et al., 2024; Oyewumi et al., 2016)	Correlates with reduced OS; siRNA targeting lowers tumor burden (Cancer Genome Atlas Research Network, 2014; Yofe et al., 2023; Okita et al., 2018; Liu et al., 2024; Oyewumi et al., 2016)
Osteosarcoma	Activates PI3K/Akt/mTOR and enhances cell proliferation/migration (qiong Ma et al., 2018; Tuo et al., 2022; Vasquez et al., 2016; Yuan and Cantley, 2008)	Associated with enhanced invasiveness; combined targeting with PI3K inhibitors shows efficacy (qiong Ma et al., 2018; Tuo et al., 2022; Vasquez et al., 2016; Yuan and Cantley, 2008)
Ovarian Cancer	Regulates glycolysis and promotes metastasis (Mouraviev et al., 2009; Tuo et al., 2024)	Acts as an independent poor prognostic factor; miR-532-3p reverses its oncogenic effects (Mouraviev et al., 2009; Tuo et al., 2024)
Renal Cell Carcinoma	Induces immune checkpoint inhibitor resistance (Ludwig et al., 2011)	Correlates with reduced progression-free survival; targeted inhibition reverses resistance (Ludwig et al., 2011)

## 6 Therapeutic frontiers: successes and challenges

Targeting GPNMB has yielded promising yet complex outcomes. Antibody-Drug Conjugates (ADCs): CDX-011 (Glembatumumab vedotin), an ADC linking an anti-GPNMB antibody to the cytotoxic agent MMAE, achieved a 17.6% objective response rate in GPNMB-high TNBC patients. However, dose-limiting toxicities like neuropathy and rash highlight the challenge of balancing efficacy and safety (Wang et al., 2021a). CR011-vcMMAE, another ADC, induced complete regression in melanoma xenografts but faces resistance due to sGPNMB shedding, which neutralizes the drug before it reaches tumors (Vaklavas and Forero, 2014). The Phase I/II NCT04561362 trial demonstrated BT8009 achieved 22.4% ORR with 60% DCR in advanced solid tumors, showing favorable safety and promising efficacy compared to traditional ADCs, particularly in TNBC and urothelial carcinoma, with ongoing dose and cohort optimization. Immune Checkpoint Combinations: Preclinical studies show that blocking GPNMB synergizes with PD-1 inhibitors. In hepatocellular carcinoma, GPNMB blockade restored CD8^+^ T-cell infiltration and enhanced anti-PD-1 efficacy (Chen et al., 2024). Small Molecule Inhibitors: mTOR inhibitors like rapamycin indirectly suppress GPNMB in lymphangioleiomyomatosis (LAM), reducing tumor growth by 60% in murine models (Gibbons et al., 2024). Emerging Technologies: CRISPR-Cas9 editing of GPNMB in glioblastoma cells reduced invasion by 70%, while nanoparticle-mediated delivery of GPNMB siRNA improved targeting and reduced off-tumor effects in lung cancer models (Tuo et al., 2024). These advances underscore the potential of precision therapies but emphasize the need to address GPNMB’s dual roles in health and disease (Table 5).

**TABLE 5:** Clinical trials of GPNMB-Targeted therapies: Efficacy outcomes.

CT ID	Phase	Intervention	Condition	Key outcomes
NCT01997333	II	CDX-011	Metastatic TNBC	17.6% ORR
NCT02302339	II	CDX-011	Melanoma	20% ORR

Against this backdrop of broader anti-tumor drug limitations, targeting GPNMB—despite its high expression in tumors—also presents notable challenges, with only its associated drug CDX-011 having entered clinical stages to date. These hurdles align with the aforementioned issues seen in major drug classes: GPNMB is not exclusive to tumor tissues, as its expression in normal tissues raises the risk of severe side effects, echoing the off-target toxicity concerns of ADCs stemming from non-cancer-specific antigen binding; additionally, GPNMB’s ECD segment is shed from tumor cells under the action of ADAM10, and this shed ECD binds to targeted drugs to block their anti-tumor activity, a problem analogous to how resistance mechanisms (like target mutations for small-molecule inhibitors) undermine drug efficacy. To address these challenges, optimizations for GPNMB-targeted therapies can be tailored to mitigate such harms—for instance, using moderate-affinity antibodies to ensure binding only to tumor tissues with high GPNMB expression, and designing administration regimens that first neutralize shed ECD in the bloodstream before delivering therapeutic agents. As research advances and drug design continues to refine these strategies, it is expected that more GPNMB-targeted candidates, along with improved iterations of ADCs and small-molecule inhibitors, will move into clinical stages and eventually find practical application.

## 7 Future directions: bridging knowledge gaps

The path forward demands innovative strategies to overcome current limitations. Biomarker Development: Serum sGPNMB levels correlate with tumor burden in gastric cancer and disease progression in lysosomal storage disorders. Validating sGPNMB as a non-invasive biomarker could revolutionize monitoring and personalized treatment Mechanistic Exploration: Elucidating GPNMB’s crosstalk with HER2 and immune checkpoints like TIGIT may unveil new combinatorial regimens. For instance, in HER2 positive breast cancer, GPNMB depletion enhanced trastuzumab efficacy by upregulating HER2 expression (Hager et al., 2022). Technological Integration: CRISPR screens and single-cell omics can map GPNMB’s interactome in tumor microenvironments, identifying context-dependent vulnerabilities. AI-driven drug design might optimize ADC structures to resist sGPNMB interference. Clinical Trials: Emerging therapeutic strategies targeting GPNMB, including CAR-T cell approaches for glioma and bispecific antibodies for NSCLC, hold potential to redefine cancer treatment paradigms. Cross-Disease Insights: Lessons from GPNMB’s role in neurodegenerative diseases, such as its neuroprotective interaction with CD44, might inspire therapies for brain metastases (Neal et al., 2018). Collaborative efforts across disciplines will be key to translating these insights into clinical breakthroughs.

GPNMB orchestrates an immunosuppressive tumor microenvironment through dual mechanisms. On the one hand, it functions as an immune checkpoint molecule that directly suppresses T cell activation and IFN-γ secretion via the DC-HIL/syndecan-4 pathway (Chung et al., 2014). On the other hand, emerging evidence reveals that GPNMB^+^TAMs in melanoma and breast cancer microenvironments exhibit significant upregulation of immunosuppressive markers including TREM2 and CD206 (Liu et al., 2024). These findings suggest that GPNMB establishes a comprehensive immune evasion network - directly through T cell inhibitory pathways while indirectly by modulating myeloid cell functions to foster immune tolerance.

Of particular significance is the spatially correlated distribution between GPNMB positive TAMs and T cell suppression phenotypes, indicating a potential positive feedback loop in tumor immune escape: GPNMB-inhibited T cells fail to effectively eliminate tumor cells, while persistently recruited and polarized GPNMB positive TAMs further exacerbate immunosuppression. This cross-regulatory network provides a theoretical foundation for developing combination targeting strategies.

## 8 Conclusion

As demonstrated in the study published in Cells (Manganelli et al., 2023), heparanase (HPSE) promotes autophagy to support glioma cell survival through structural foundations of lysosomal enrichment and autophagosome localization, combined with its enzymatic activity of cleaving heparan sulfate to remodel the extracellular matrix and non-enzymatic activity of regulating intracellular signaling pathways. Its novel inhibitor, RDS 3337, can penetrate the cell membrane, inhibit the autophagy-promoting function of intracellular HPSE, thereby blocking autophagic flux and inducing apoptosis in U87 glioblastoma cells. Given that GPNMB is also involved in autophagy regulation and tumor cell survival in gliomas, the mechanism of HPSE action in this study can provide insights for subsequent investigations into whether GPNMB exerts its effects through a similar “specific subcellular localization + dual activity” mode and whether it is associated with core autophagic pathways such as MTORC1. Additionally, it offers directions for the development of GPNMB-targeted intervention strategies and the exploration of the potential of combined intervention targeting both HPSE and GPNMB.

The therapeutic targeting of GPNMB presents a fundamental biological conundrum, as this multifunctional glycoprotein plays essential roles in maintaining neural myelin homeostasis and osteogenesis while concurrently driving tumor immune evasion and metastatic progression. Emerging research has revealed that GPNMB-mediated regulation of myelin clearance mechanisms contributes to neural repair processes, directly accounting for the dose-limiting neurotoxicity observed in approximately 20% of treated patients (Kloosterman et al., 2024). Overcoming this therapeutic challenge requires elucidation of tumor-specific conformational epitopes, development of biomarker-guided monitoring systems, and engineering of tissue-selective delivery platforms.

From a translational perspective, mechanistic insights into GPNMB’s involvement in metabolic reprogramming and receptor crosstalk provide critical opportunities for novel combination therapies. The convergence of next-generation ADC technologies with advanced organoid models now enables the development of functionally selective targeting systems. This therapeutic innovation necessitates systematic integration of foundational neuroscience discoveries with clinical translation in oncology, establishing a multidisciplinary framework that may extend to other therapeutically relevant targets exhibiting comparable functional duality. Such an approach exemplifies the evolving paradigm of precision medicine in cancer therapeutics.
